# Evaluation of awareness and performance towards COVID-related disinfectant use among the university communities in Lebanon

**DOI:** 10.1186/s12889-023-16515-9

**Published:** 2023-08-18

**Authors:** Wissam Ghach, Jihan Safwan, Racha Kerek, Nisreen Alwan

**Affiliations:** 1 Faculty of Communication, Arts and Sciences, Canadian University Dubai, Dubai, United Arab Emirates; 2 School of Health Sciences, Modern University for Business and Science, Beirut, Lebanon; 3 School of Pharmacy, Lebanese International University, Beirut, Lebanon; 4 INSPECT-LB, Institut National de Santé Publique d’Épidémiologie Clinique et de Toxicologie-Liban, Beirut, Lebanon; 5 Faculty of Health Sciences, Lebanese University, Tripoli, Lebanon; 6 Department of Natural Sciences, Lebanese American University, Byblos, Lebanon; 7 College of Health Sciences, Abu Dhabi University, Abu Dhabi, United Arab Emirates

**Keywords:** COVID-19, Awareness, Performance, Disinfectant Use, University community, Lebanon

## Abstract

The fast spread of COVID-19 reinforced the daily use of disinfectants around the world. However, the awareness gap of disinfectant use could lead to health risks during the prevention of the pandemic. This study aims to assess the level of awareness and performance towards COVID-related disinfectant use among various university communities (student, staff, and faculty) in Lebanon. Methods: A cross-sectional study was conducted between December 2021 and June 2022 among 925 participants (males and females aged between 18 and 64 years old) from academic settings in Lebanese universities using convenience sampling. An online validated survey (score-based questionnaire) of personal disinfectants’ utilization was conducted to evaluate the awareness and performance levels using SPSS (version 21). Mann–Whitney and Kruskal–Wallis tests were used to check significant differences in awareness and performance levels among gender, age, provinces, educational level, university status, and field of study. Friedman test was used to test for significant differences in performance level questions pre-and post-COVID-19. The Spearman correlation test was used to determine the correlation between the awareness and performance of the respondents regarding the use of disinfectants. Results: It was found that the majority of the respondents showed a weak level of awareness (70.8%) while their performance (61.9%) was moderate. Spearman’s correlation analysis concluded a weak correlation between the awareness and performance levels (*p* < 0.01). The Mann–Whitney test indicated that there was a significant difference (*p* < 0.05) in awareness and performance levels between males and females. Another notable variable was the educational level of the respondents (*p* < 0.05) with postgraduate degrees holders recording higher mean scores of awareness than the ones with undergraduate and high school degrees as per Kruskal–Wallis test. Significant differences were shown in awareness scores among the age groups and in performance scores among the field of study (*p* < 0.05). Conclusions: The study findings highlighted the necessity of awareness campaigns and training programs addressing the technical handling of disinfectants among the communities in Lebanon. Lebanese governmental authorities (Ministry of Public Health, MoPH, and Ministry of Education and Higher Education, MEHE), and the healthcare professionals and public health researchers in Lebanon may utilize this new evidence to initiate public health interventions as a part of the United Nations (UN) sustainability goal of wellbeing (Sustainable Development Goal 3, SDG 3).

**Supplementary Information:**

The online version contains supplementary material available at 10.1186/s12889-023-16515-9.

## Background

On the 8^th^ of December 2019, an incidence of viral infection caused by a new coronavirus named “Severe Acute Respiratory Syndrome Coronavirus-2 (SARS-CoV-2)” was reported in Wuhan, China causing mild to severe respiratory illness and death [1–3]. The SARS-CoV-2 or COVID-19 (Coronavirus Disease 2019) disease has spread quickly globally (82,294 confirmed and 2804 death cases among 46 countries), and as a consequence, it was classified as a public health cataclysmic pandemic by the World Health Organization (WHO) on March 11^th^, 2019 [4]. The mode of COVID-19 transmission was identified by direct human-to-human interactions (skin infectivity of living and deceased organisms on the time scale of days) and human-to-object interactions when the solid surfaces covered by viable SARS-CoV-2 from respiratory droplets and aerosols of the infected people remain infectious on the timescale of days under ambient indoor conditions [5–7]. To reduce the transmission of COVID-19, WHO has recommended several preventive measures in public areas such as wearing a mask, keeping an appropriate distance from others, washing hands, and utilizing chemical disinfectants after touching objects in public facilities [7].

To limit the adverse effects of the use of chemical disinfectants (e.g., ethanol, isopropanol, chlorine-containing disinfectants, benzalkonium chloride, quaternary ammonium compounds, and sodium hypochlorite), the Centers for Disease Control and Prevention (CDC) recommended guidelines for cleaning and disinfection [8, 9]. Methanol, for example, has the weakest antimicrobial effect as compared to other alcohols and may cause birth defects in the central nervous system and inflammation of the eye (visual failure). Thus, it is not recommended for the disinfection process [10, 11]. As an alternative to methanol, CDC recommends ethanol (70%) and isopropanol (70%) due to their low levels of toxicity and high antimicrobial effects [10]. The antimicrobial mechanism is explained by the denaturation of microbial and viral proteins as well as by the inhibition of the production of metabolites essential for rapid cell division. As a result, these alcohols act as a potent virucidal agent that inactivates lipophilic and hydrophilic viruses [10]. However, isopropanol and ethanol may also lead to illness when misused causing severe depression in the respiratory or central nervous system, respiratory arrest, cardiac arrest, and hypotension [12, 13]. Chlorine compounds (also known as hypochlorite) are classified with a broad spectrum of bactericidal activity, as a cheap and fast-acting product as well as being unaffected by the hardness level of tap water. Sodium hypochlorite at the household concentration level (5.25–6.15%) can produce ocular irritation or oropharyngeal, esophageal, and gastric burns among users [10]. Besides, hypochlorite (bleach) shall be applied without mixing with acids, amines, any other detergents to avoid the occurrence of acute lung diseases and cancer [14]. For example, the accidental mixing of bleach with ammonia or acids releases toxic chloramine and chlorine gases, respectively which in turn may cause severe eye and throat burning, breathing difficulties, and even death. Moreover, the accidental mixing of bleach with rubbing alcohols releases chloroacetone and chloroform which in turn may cause damage to the nervous system, eyes, lung, skin, liver, kidneys [15, 16].

Since 2020, the massive release of chemical-based disinfectants to the environment may be considered as double-edged sword where disinfectants kill the SARS-CoV-2 virus but also impose health risks and environmental stress by affecting other non-target organisms (virus, plants, microorganisms) in a dose-dependent manner, and finally promoting traits of drug resistance human pathogens [17]. To safely prevent COVID-19 infections and to avoid any potential harmful effects on the environment and public health, there is a need for increased public awareness on the use of disinfectants. As educational settings include frequent interactions among large numbers of people providing a suitable milieu for the spread of pathogens, adopting appropriate awareness and performance regarding disinfectants use are vital in educational institutions [18–22]. Several studies have focused on the awareness and performance regarding preventive measures among university students [19–22]. However, these studies have investigated awareness related to COVID-19 transmission, diagnosis, quarantine, and general preventive measures (e.g., mask use, handwashing, physical distancing, and the frequent disinfection of the human body and surfaces). To the best of our knowledge, no published studies had investigated the awareness and performance regarding the use of chemical disinfectants among the university community in the Middle Eastern Arab countries especially in those suffering from harsh financial, economic, and healthcare crises such as Lebanon [23, 24].

During the onset of the pandemic, a high incidence of COVID-19 was expected in the Middle Eastern Arab communities as a result of religious practices (e.g., pilgrimages and gathering for common prayer in mosques and churches) and humanitarian quality of refugee camps in the Middle Eastern countries including Lebanon [25]. In the latter, investigations of the public awareness and performance regarding the use of COVID-related disinfectants would be necessary during the fast spread of COVID-19, especially given the deep economic crisis and the harsh shortage in the Lebanese healthcare system (healthcare professionals, surgical equipment, materials, and pharmaceuticals) [26, 27]. Despite the unfavorable expectations, limited studies have investigated the quality of awareness and practices of these communities regarding the chemical-based disinfectant use for safe and effective prevention of SARS-CoV-2. For example, a single study focused on the awareness, preference, and adherence of the public community to the use of several types of hand sanitizers in Lebanon [28]. Almost half of the sample population in this study reported skin irritation after the use of hand sanitizers (gel, liquid, and wet wipes). On the other hand, an Iranian study investigated public awareness and performance regarding the safe use of disinfectants [29]. They found that 52% of the participants had a weak level of awareness while 56% had a good level of performance. Applying such a study is highly recommended to evaluate the safe prevention of COVID-19 and to limit the impact of absenteeism on academic performance in the Middle East, especially among the university communities of Lebanon.

According to the statistics published by the Lebanese Ministry of Public Health (MoPH), over 1 million confirmed cases of COVID-19 and 10,000 deaths have been reached in Lebanon [30]. Since the announcement of the pandemic, the ministry has enforced the WHO preventive measures (physical distancing, wearing mask, and regular hygiene practices) to limit the spread of COVID-19 and its impact on public health in Lebanon. However, the Lebanese communities should be fully aware on how to safely use the chemical-based disinfectants for effective prevention of the pandemic. To the best of our knowledge, the level of awareness and performance toward the safe use of COVID-related disinfectants in Lebanon has not been evaluated based on the guidelines of the CDC and WHO. For the first time, this paper aims to assess the level of awareness and performance regarding disinfectant use among university communities in Lebanon. The outcome is expected to reduce the knowledge gap and reinforce safe practices of preventive measures through awareness interventions at Lebanese universities. Additionally, the outcome is expected to enrich the governmental authorities, healthcare professionals and public health researchers with new evidence on the awareness level of the safe use of COVID-related disinfectants, and their potential implications on the public health at the academic institutions in the developing countries.

## Methods

A cross-sectional study was conducted between December 2021 and June 2022 to assess the awareness and performance levels regarding the use of chemical disinfectants in Lebanese universities (Middle Eastern). An online survey was distributed using convenience sampling throughout the social media platforms and through emails via the research departments of the Lebanese universities.

### Population

A total of 925 individuals aged more than or equal to 18 years old from various Lebanese universities participated electronically in this study. Children (aged less than 18 years old) and non-registered respondents at one of the Lebanese universities are excluded from this study. The subject population was stratified into groups of gender, age, university department, study program, and the respondent’s status at the academic institution (staff, faculty, or student).

### Study tool

The validated questionnaire that was used, has been adapted from the Iranian study of disinfectants (supporting information) [29]. The profile section of the questionnaire included eight items about age, sex, educational level, province, university status (student, staff, or faculty), university department, study program, and experience with SARS-CoV-2 infection. The source of COVID information section included several choices of resources such as personal experience, internet webpages, television (TV), social media platforms, healthcare professionals, the Lebanese MoPH, and webpages of CDC and WHO. The awareness and performance sections included 10 True–False items and 18 Yes/No and Likert-type items (never, rarely, sometimes, most of the time, always) used to measure the quality of knowledge and health performance regarding proper and timely use of antiseptic agents among the study population. Awareness items were graded according to the following scores: 0 (incorrect response) and 1 (correct response). The mean score of awareness was then divided into intervals: weak [0, 4], moderate [5,7], and good [8-10]. Performance items were graded according to the following scores: 0 (no and never), 1 (yes and rarely), 2 (sometimes), 3 (most of the time), and 4 (always). The mean score of performance was then divided into intervals: weak [0, 20], moderate [21, 40], and good [41–57].

### Study analysis

Data was analyzed using the Statistical Package for the Social Sciences, version 21 (SPSS). To reflect the sociodemographic profile of respondents, percent frequency was obtained and organized in tables. Median with interquartile range (IQR) were also calculated and tabulated to summarize the community levels of awareness and performance. Normality and homogeneity of variances were tested using Kolmogorov–Smirnov and Levene’s tests respectively for all variables rendering violations in these two assumptions with *p* < 0.05. For that purpose, Mann–Whitney test or Kruskal–Wallis test was used instead of a t-test or One-way ANOVA. Mann–Whitney test was used to determine if significant differences exist in awareness and performance levels with regard to gender. Kruskal–Wallis test was used to check for differences in awareness and performance for the rest of variables. Friedman test was used to test for significant differences in performance level questions pre-and post-COVID-19. To determine the correlation between awareness and performance of respondents regarding the use of disinfectants, Spearman correlation test was used. A multinomial logistic regression was used to examine if all variables mentioned above (gender, age, provinces, educational level, university status, and field of study) were associated with the levels of awareness and performance. All data analysis was carried out at a significance level of 0.05 (Confidence intervals at 95%) with values of *p* < 0.05 being statistically significant.

### Ethical considerations

The study protocol was approved by the Institutional Review Board (IRB) at the Modern University for Business and Science (MUBS) (MU20210924-25). The first page of the online google survey included an informed consent to identify the purpose, risks, benefits, and confidentiality of the study. Additionally, the informed consent was concluded by a statement stressing that participation is voluntary, and that the submission of the questionnaire indicates the consent of the individual to participate in the study.

## Results

In this study, the majority of the study respondents were females (64.5%) with a sex ratio (M:F) of 1:1.82 and a mean age of 24.43 (± SD = 7.76) years. Most of the respondents (85.4%) were residents of the five Lebanese provinces (Beirut, Mount Lebanon, Beqaa, South Lebanon, and North Lebanon). In terms of educational level, most of the study population were students (78.4%) at the undergraduate level (80.4%). The age range (18–29 years), followed by (30–39 years) represented most of the study population by percentages of 80.6, and13.1, respectively (Table 1).

**Table 1:** Frequencies and percentages of the study population characteristics at the Lebanese universities during the spread of COVID-19

Variables	Number	Percentage
**Gender**		
Male	328	35.5%
Female	597	64.5%
**Age**		
18–29	745	80.6%
30–39	121	13.1%
40–49	44	4.8%
50–64	14	1.51%
**Provinces**		
Beirut	203	21.9%
Mount Lebanon	201	21.7%
Beqaa	126	13.6%
North Lebanon	138	14.9%
South Lebanon	125	13.5%
Baalbek – Hermel	25	2.7%
Akkar	50	5.4%
Nabatieh	57	6.2%
**Educational Level**		
High school	188	20.3%
Bachelor’s degree	556	60.1%
Master’s degree	148	16%
PharmD and Ph.D. degrees/Postdoctoral fellowship	33	3.5%
**University Status**		
Student	808	87.4%
Staff	37	4%
Faculty	80	8.6%
**Field of Study**		
Health sciences (nutrition, nursing, public health, and optometry)	84	9.1%
Natural sciences (chemistry, biology, physics, math, and biochemistry)	81	8.8%
Medical sciences (biomedical sciences, medical lab, and biomedical engineering)	58	6.3%
Pharmacy	24	2.6%
Computer sciences (information technology, computer science, computer engineering)	97	10.5%
Engineering (electrical, industrial, electronic, and mechanical engineering)	38	4.1%
Education and social sciences (child education, social work, teaching English, and translation)	127	13.8%
Art and mass communication (graphic design, interior design, radio, and TV)	37	4%
Business (Tourism, management, hospitality, marketing, and human resources	154	16.6%
Staff (Administration, human resources, public relations, and information technology)	37	4%
Missing data	188	20.3%

**Fig. 1:**
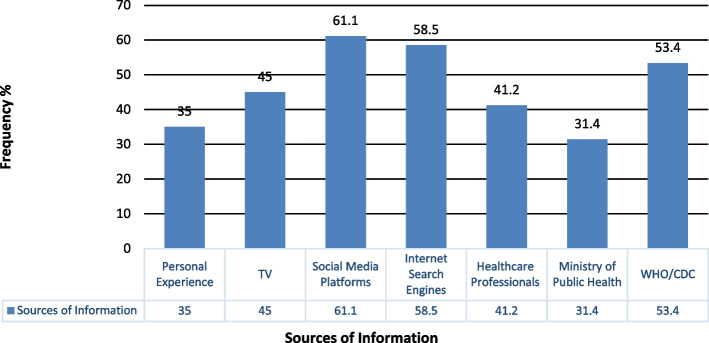
Bar graph representing the community's reliance (frequency %) on different resources of COVID-19 information

Looking at the sources of COVID-19 information among the study population (Fig. 1), respondents mostly preferred social media platforms (61.1%) and internet search engines (58.8%) to get their awareness during the prevention of the pandemic. They showed the lowest reliance on the governmental resource authority (Lebanese MoPH: 31.4%) and personal experience (35%).

### Evaluation of awareness (A) and performance (P) 

The evaluation of the community awareness regarding disinfectants is represented in Table 2. The awareness of personal disinfection was somehow low where almost half of the study population had no information about the choice of alcohol (A1: 49.7%) and its concentration (A8: 48.5%) for the effective disinfection against SARS-CoV-2. Furthermore, half of the study population did not identify methanol as a toxic and deadly alcohol (A3: 55.8%). Similar to personal disinfection, the majority of the respondents had no information on how to disinfect food and vegetables with the water-vinegar mixture (A6: 58.6%; A7: 59.7%). Awareness about the disinfection of solid surfaces was also very alarming as most of the study population had no information on how to prepare (A10: 79.2%; A5: 91.5%) and use a chlorine solution (A9: 86.4%). On the other hand, the findings of community performance showed that 98.1% of respondents washed their hands immediately upon arrival home during the pandemic (P1) compared to 88% before the pandemic (P2). Regarding the implementation of personal hygiene during the pandemic, a variation in the community’s commitment was recorded as follows: washed hands or used gloves when buying bread (P4: 62.4% compared to 31.4% before the pandemic: P5), touched eyes and face with disinfected hands (P6: 49.3% compared to 60.3% before the pandemic: P7) and washed hands properly for at least 20 s (P9: 92.6% compared to 73.6% before the pandemic: P10). Focusing on the food safety protocols, 65.5% of the study population washed fruit and vegetables with special disinfectants (P16) during the outbreak as compared to 51.4% before the pandemic (P17). Furthermore, Friedman test revealed significant statistical differences (*p* < 0.001) between pre-and post-COVID-19 for questions (P1 vs. P2), (P4 vs. P5), (P6 vs. P7), (P9 vs. P10), (P16 vs. P17).

**Table 2:** Frequencies and percentages of the respondents’ awareness regarding the use of disinfectants

Awareness	Correct Responses	
	**Frequency**	**Percentage**
**A1**—Which alcohol is used as a disinfectant? (**Ethanol**, Methanol, Both, None, Don’t Know)	465	50.3%
**A2**—Which one is used for surface disinfection? (Sodium hypochlorite, Perchlorine, Alcohol, **All of the above**, Don’t Know)	144	15.6%
**A3**—Which alcohol is industrial alcohol that is toxic and deadly? (Ethanol, **Methanol**, Ethanol & Methanol, None, Don’t Know)	409	44.2%
**A4**—How much chlorine is normally present in bleach? **(5%,** 20%, 70%, 100%, Don’t Know)	360	38.9%
**A5**—What is the ratio of bleach to water for making surface disinfection? (1 to 2, 1 to 5, 3 to 1, **1 to 50**, Don’t Know)	88	9.5%
**A6**—For pre-disinfection of fruits and vegetables, how many minutes do they need to be in water and vinegar? (2 to 5, **5 to 15**, 30, 60, Don’t Know)	383	41.4%
**A7**—What is the recommended ratio of vinegar to water for pre-disinfection of fruits and vegetables? (**1 to 3**, 7 to 10, 15 to 20, 20 to 30, Don’t Know)	373	40.3%
**A8**—Which one is the most effective concentration of alcohol for skin disinfection? (0.5%, 1%, **70%,** 95%, Don’t Know)	476	51.5%
**A9**—How long can it take for the disinfectant solution prepared by chlorine to be used for disinfection? (1 h, **1 day**, 1 week, 1 month, Don’t Know)	126	13.6%
**A10**—At which temperature do you use water to dilute disinfectant solution? (**Room-temperature**, Warm, Hot water, None, Don’t Know)	192	20.8%

^*^Options in bold indicate correct answers

**Fig. 2:**
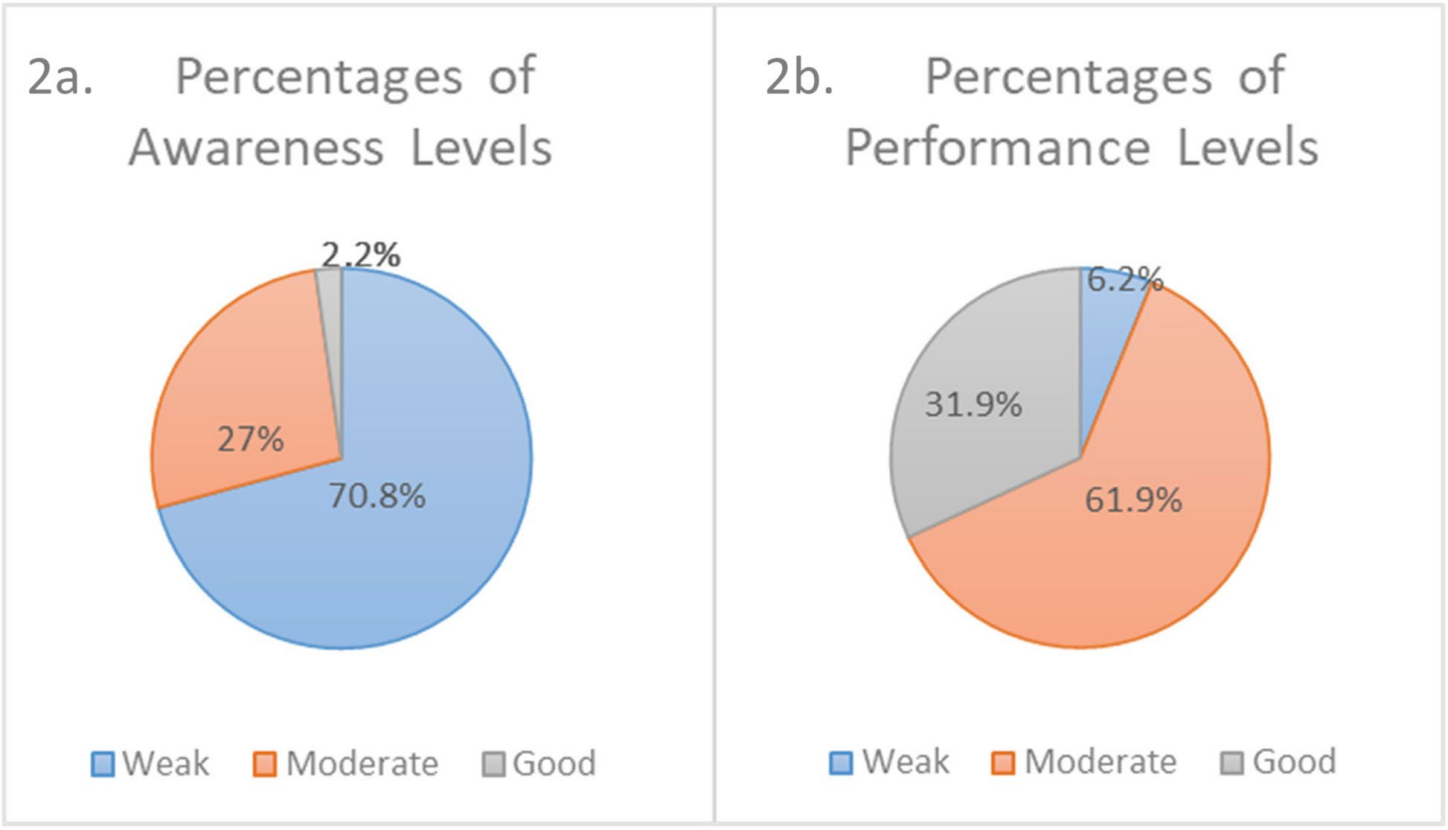
Pie charts representing the % frequency of community levels of awareness (**a**) and performance (**b**)

Among the study population, only 29.2% of the sample population (270 out of 925 respondents) showed moderate-to-good awareness about disinfectants (Fig. 2a). On the other hand, 93.8% of the sample population (868 out of 925 respondents) showed moderate-to-good performance levels as shown in Fig. 2b. The Spearman correlation test showed that the community awareness and performance were weakly correlated (rho = 0.14, *p* < 0.05). The results of the likelihood ratio tests using multinomial logistic regression showed that only gender has significant association with the level of awareness (χ^2^ = 8.682, *p* = 0.013 < 0.05) and performance (χ^2^ = 35.16, *p* < 0.001).

### Association of awareness and performance with the study population’s characteristics 

In Table 3, a comparative analysis between the study population characteristics and their levels of awareness and performance concerning the use of disinfectants is represented. Mann–Whitney test indicated that there were significant differences (*p* < 0.05) in awareness and performance levels between males and females, where females recorded higher median scores of performance (37 with IQR of 14). Another notable variable was the educational level of the study population where the respondents with postgraduate degrees (master’s degrees, PharmD degrees, Ph.D. degrees, and postdoctoral fellowships) recorded higher median scores of awareness (Table 3). This is in agreement with the Kruskal–Wallis test that indicated a significant difference (*p* < 0.05) between respondents with post-graduate degrees and undergraduate/high school degrees. On the other hand, the respondents with Bachelor’s degrees (undergraduate university level) showed a higher median score of performance (37 with IQR of 12) than the other groups. The other variables (age, provinces, field of study, and university status) showed no significant differences in awareness and performance (*p* > 0.05) except for the evaluation of awareness scores among the age groups and performance score among the field of study (*p* < 0.05).

**Table 3:** Statistical association of awareness and performance levels with study population characteristics

Variables	Awareness			Performance		
	**Median**	**IQR**	*p*-value^*^	**Median**	**IQR**	*p*-value^*^
**Gender**						
Male	3.00	3.00	**0.001**	34	14	**< 0.001**
Female	3.00	3.00		37	14	
**Age**						
18–29	3.00	4.00	**0.03**	36.00	13.00	0.75
30–39	3.00	3.00		36.00	16.00	
40–49	4.00	4.00		34.50	16.00	
50–64	3.00	3.00		36.00	14.00	
**Provinces**						
Beirut	3.00	4.00	0.31	36.00	14.00	0.26
Mount Lebanon	3.00	4.00		37.00	13.00	
Beqaa	4.00	3.00		37.00	16.00	
North Lebanon	3.00	3.00		36.00	13.00	
South Lebanon	3.00	3.00		34.00	14.00	
Baalbek – Hermel	3.00	4.00		34.00	11.00	
Akkar	3.00	2.00		35.00	13.00	
Nabatieh	3.00	3.00		37.00	18.00	
**Educational level**						
High School degree	3.00	3.00	**< 0.001**	35.00	15.00	0.075
Bachelor’s degree	3.00	3.00		37.00	12.00	
Master’s degree	4.00	3.00		35.00	15.00	
PharmD, Ph.D. degrees and postdoctoral fellowship	4.00	4.00		34.00	16.00	
**University status**						
Student	3.00	3.00	0.21	36.00	14.00	0.25
Staff	3.00	3.00		38.00	14.00	
Faculty	3.50	4.00		33.50	13.00	
**Field of study**						
Pharmacy, health, medical, natural sciences	3.00	3.00	0.94	37.00	14.00	**0.01**
Other fields of study	3.00	3.00		35.00	14.00	
Staff	3.00	4.00		37.00	13.00	
Missing	3.00	3.00		36.00	14.00	

^*^Values in bold show significant *p*-values less than 0.05

## Discussion

For effective control of the pandemic spread in the Lebanese academic institutions, this study was conducted to shed light on the levels of awareness and performance of university communities regarding the safe use of disinfectants. To date, no similar studies have been published in Lebanon. An anticipated output of this study is to provide the Lebanese governmental authorities (MoPH, and MEHE), and the healthcare professionals and public health researchers in Lebanon with new evidence on the use of COVID disinfectants and their potential implications on the community health. The study findings also provide a baseline to initiate public health interventions to raise awareness of the safe use of disinfectants and consequently limit the adverse effects of chemical misuse among developing countries as a part of the United Nations (UN) sustainability goal of wellbeing (Sustainable Development Goal 3, SDG 3).

Worldwide, public awareness about the preventive measures and the safe prevention against COVID-19 are matters of concern. To benefit from the public use of the internet during remote learning and working, governmental and non-governmental organizations (national and international) have conducted several awareness campaigns about COVID-19 prevention [31, 32]. Based on this study, people preferred to get their information from healthcare professionals and the Lebanese MoPH, internet webpages (including WHO and CDC), and social media platforms. However, the internet webpages and social media platforms may add a new concern regarding the unverified knowledge and misleading information that could misguide people during the prevention of the pandemic [33–35]. Additionally, most of the online resources were mostly limited to providing the basic knowledge of preventive measures (physical distancing, mask, and disinfectant use) rather than providing the technical protocols of disinfectant handling (e.g., protocols of dilution, storage conditions, types of chemicals) [21]. On the other hand, some electronic resources used artificial intelligence applications to trace contacts and movement as well as to enforce quarantine compliance and symptom checking which could represent a serious threat for privacy protection among the populations [36]. Thus, to enhance the role of information resources on public awareness, proper training and awareness campaigns should be developed virtually and on-site (municipalities, academic institutions, industries and companies, and shopping centers).

Focusing on the geographical location of Lebanon in Middle East (belonging to the Arabic culture), the community was expected to show similar levels of awareness and performance towards the hygiene-based preventive measures of COVID-19 compared to the studies of four Arabic speaking Middle Eastern countries (Jordan, Kuwait, Saudi Arabia, and United Arab Emirates) where participants embraced misconceptions about COVID-19, resulting in ineffective protective measures against SARS-CoV-2 infection [37, 38]. However, in these studies, most of the study population showed a higher level of performance than awareness. This is in congruence with an Iranian study [21]. The finding could be explained by the structural design of the survey tool of the studies. The awareness questions of the tool had a technical nature (type of active ingredient of disinfectants, nature of toxicity, the optimum concentration of use, optimum temperature of preparation); however, performance questions had the basic and scientific level of preventive measures (hand wash, disinfection of fruits and vegetables, disinfection of solid surfaces and packaging, glove use, and separate use of detergents). Furthermore, the tool successfully compared the community performance before and during the pandemic to shed the light on the importance of awareness to raise the participant's adherence to the most recommended practices for COVID-19 prevention.

Similar to other studies [21, 28, 29, 39], females recorded higher performance scores than males. The finding may be explained by the fact that women are primarily responsible for taking care of the home and using disinfectants to maintain their own and their families' health [40]. Another notable variable was the impact of education level. In agreement with the literature, the higher the education level, the higher the level of awareness and performance concerning preventive measures and disinfectant use [20, 29]. Unexpectedly, the university status showed no significant difference in awareness and performance levels among students, faculty, and staff. Unlike other studies, the field of study (health, pharmacy, medical, and natural sciences vs other fields) did not enhance the mean scores of awareness and performance among the groups of the study population [22].

### Study weakness and limitations

The study findings may include several limitations such as result bias which could derive from the self-responding tool and the unequal distribution of the respondents (dominant number of students and females). An additional limitation was the online data collection during the pandemic which could weaken the randomness of the study sampling. To overcome the limitations of the online data collection where potential respondents (mostly students) were expected to be users of the social media platforms, the e-survey was also distributed among the academic communities (student, staff, and faculty) via the institutional research offices at the Lebanese universities.

## Conclusions

The current study revealed that the university communities in Lebanon had a higher performance level (moderate-to-good: 93.8%) than awareness (moderate-to-good: 29.2%) with a weak correlation between the awareness-performance variables highlighting the necessity of awareness campaigns to maintain high levels of awareness and performance specifically regarding the technical handling of disinfectants. The findings showed a positive shift in the communities’ behavior regarding personal hygiene during the pandemic as compared to before COVID-19. Associated variables such as gender, age, and educational level contributed positively to awareness and/or performance levels among the university communities where females, highly educated (postgraduate degrees holders), and middle-aged (40–49 years old) respondents recorded higher scores of awareness and/or performance. These findings include some research bias (self-responding, weak random sampling, and unequal distribution of the respondents). Interventions, future risk assessment and toxicological studies are recommended to address the improper use of chemical-based disinfectants in the developing countries such as Lebanon. Additionally, conducting awareness campaigns and educational programs on the safe use of chemical-based disinfectants and evaluating the effectiveness of these interventions in improving community awareness and performance levels are highly recommended at the academic and household levels especially in the developing countries.

### Supplementary Information

**Additional file 1:** **Supplementary Materials.** Include questionnaire items with point scores.

## Data Availability

The datasets used and/or analysed during the current study are available from the corresponding author on reasonable request.

## Abbreviations

COVID-19: Coronavirus Disease 2019
WHO: World Health Organization
CDC: Centers for Disease Control and Prevention
MoPH: Ministry of Public Health
MEHE: Ministry of Education and Higher Education
UN: United Nations
SDG: Sustainable Development Goal
