# Supplementary material for: Evaluation of awareness and performance towards COVID-related disinfectant use among the university communities in Lebanon
Source: BMC Public Health. 2023 Aug 18;23:1582. doi: 10.1186/s12889-023-16515-9 (PMC10439643; doi:10.1186/s12889-023-16515-9)
Supplement: Supplementary file 1 — Additional file 1: Supplementary Materials. Include questionnaire items with point scores. [file 12889_2023_16515_MOESM1_ESM.docx]

Supplementary Material

Evaluation of awareness and performance towards COVID-related disinfectant use among the university communities in Lebanon

Wissam Ghach^1,2*^, Jihan Safwan^3,4^, Racha kerek^5,6^ and Nisreen Alwan^7*^

* Correspondence: Wissam Ghach, Ph.D.; [wissam.ghach@cud.ac.ae](mailto:wissam.ghach@cud.ac.ae);

Nisreen Alwan, Ph.D.; nisreen.alwan@adu.ac.ae

# Supplementary Data (Study tools)

**Section 1 - Awareness:**

- - - 1. Which alcohol is used as a disinfectant? (**Ethanol**, Methanol, Both, None, Don’t Know)
      2. Which one is used for surface disinfection? (Sodium hypochlorite, Perchlorine, Alcohol, **All**, Don’t Know)
      3. Which alcohol is industrial alcohol that is toxic and deadly? (Ethanol, **Methanol**, Both, None, Don’t Know)
      4. How much chlorine is normally present in bleach? (100%, 70%, 20%, **5%**, Don’t Know)
      5. What is the ratio of bleach to water for making surface disinfection? (1 to 5, 1 to 2, 3 to 1, **1 to 50**, Don’t Know)
      6. For pre-disinfection of fruits and vegetables, how many minutes do they need to be in water and vinegar? (5 to 15, **2 to 5**, 60, 30, Don’t Know)
      7. What is the recommended ratio of vinegar to water for pre-disinfection of fruits and vegetables? (**1 to 3**, 7 to 10, 15 to 20, 20 to 30, Don’t Know)
      8. Which one is the most effective concentration of alcohol for disinfection? (95%, **70%,** 0.5%, 1%, Don’t Know)
      9. How long can it take for the disinfectant solution prepared by chlorine to be used for disinfection? (**24 hour**, 1 hour, 1 week, 1 month, Don’t Know)
      10. At which temperature do you use water to dilute disinfectant solution? (Warm, **Cold**, Hot water, None, Don’t Know)

**Score: 1 point for each correctly answered question (bolded).**

**Section 2 - Performance:**

| 1. Do you wash your hands when you get home?   Always (4) Most of the time (3) Sometimes (2) Rarely (1) Never (0) |
| --- |
| 1. Did you use to wash your hands when you got home (before the COVID-19 pandemic)?   Yes (1) No (0) |
| 1. Do you disinfect keys, cards, mobile phones, and equipment used outside when you get home?   Always (4) Most of the time (3) Sometimes (2) Rarely (1) Never (0) |
| 1. Do you disinfect your hands or use gloves when buying items such as bread?   Always (4) Most of the time (3) Sometimes (2) Rarely (1) Never (0) |
| 1. Did you use to disinfect your hands or use gloves when buying items such as bread (before the COVID-19 pandemic)?   Yes (1) No (0) |
| 1. Do you touch your face or eyes with uncontaminated hands when you are outdoors?   Always (4) Most of the time (3) Sometimes (2) Rarely (1) Never (0) |
| 1. Did you use to touch your face or eyes with uncontaminated hands when you were outdoors (before the COVID-19 pandemic)?   Yes (1) No (0) |
| 1. Do you disinfect your house and work surfaces?   Always (4) Most of the time (3) Sometimes (2) Rarely (1) Never (0) |
| 1. Do you wash your hands carefully and correctly (at least for 20 seconds, all over your hands and wrists)?   Always (4) Most of the time (3) Sometimes (2) Rarely (1) Never (0) |
| 1. Did you use to wash your hands carefully and correctly (before the COVID-19 pandemic)?   Yes (1) No (0) |
| 1. Do you disinfect door handles?   Always (4) Most of the time (3) Sometimes (2) Rarely (1) Never (0) |
| 1. Do you disinfect the exterior and interior of the handles, steering wheel, and gear lever of your car?   Always (4) Most of the time (3) Sometimes (2) Rarely (1) Never (0) |
| 1. Do you disinfect purchased items containing packaging?   Always (4) Most of the time (3) Sometimes (2) Rarely (1) Never (0) |
| 1. Do you get alcohol and disinfectants from reputable pharmacies and shopping centers?   Always (4) Most of the time (3) Sometimes (2) Rarely (1) Never (0) |
| 1. Do you wash your hands after opening un-disinfected packages?   Always (4) Most of the time (3) Sometimes (2) Rarely (1) Never (0) |
| 1. Do you use special disinfection solutions to disinfect fruit and vegetables?   Always (4) Most of the time (3) Sometimes (2) Rarely (1) Never (0) |
| 1. Did you use to apply special disinfection solutions to disinfect fruit and vegetables (before the COVID-19 pandemic)?   Yes (1) No (0) |
| 1. Do you disinfect using separate cleaners (without combining cleaners and bleaches)?   Always (4) Most of the time (3) Sometimes (2) Rarely (1) Never (0) |
